# Psychoemotional distress on patients with cutaneous manifestations and SARS-CoV-2 infection

**DOI:** 10.1192/j.eurpsy.2023.1652

**Published:** 2023-07-19

**Authors:** C. Sapaniuc, M. Rascanu, D. Bran, G. A. Lacatusu, F. M. Rosu, M. Pulbere, L. Gheuca-Solovastru, D. C. Manciuc

**Affiliations:** 1 Infectious Diseases, “Sf. Paracheva” Infectious Diaseases Clinical Hospital; 2 Infectious Diseases, “Grigore T. Popa” University of Medicine and Pharmacy; 3 Psychologist; 4 anesthesia and intensive care, “Sf. Paracheva” Infectious Diaseases Clinical Hospital; 5 anesthesia and intensive care, “Grigore T. Popa” University of Medicine and Pharmacy; 6 dermatologist, “Sf. Paracheva” Infectious Diaseases Clinical Hospital; 7 dermatologist, “Grigore T. Popa” University of Medicine and Pharmacy; 8 dermatologist, “Sf. Spiridon” Emergency Hospital, Iasi, Romania

## Abstract

**Introduction:**

The new coronavirus had a huge impact on individuals from a social, economical and psychological point of view. In march 2020 The World Health Organization declared the coronavirus diseases 2019 (COVID-19) a pandemic and highlighted its psychoemotional implications.

**Objectives:**

The aim of this study was to investigate the psychological distress during the COVID-19 pandemic in patients with cutaneous manifestations and SARS-CoV-2 infection.

**Methods:**

Data was collected from one first-line infectious diseases hospital from northeastern Romania. All patients with cutaneous manifestations and SARS-CoV-2 infection who accepted to answer the quality of life questionnaire (QLQ) were included in the study. The descriptive data analysis was conducted with the SPSS.

**Results:**

A total of 191 patients with cutaneous manifestations and COVID-19 completed a questionnaire concerning their psychological distress and financial burden during the pandemic. Patients with chronic dermatological diseases (psoriasis) – 31% reported the highest level of psychological distress and financial burden compared to patients with acute cutaneous manifestations (chickenpox, dermatitis, infectious mononucleosis, steven-johnson syndrome, etc.). Furthermore, this patients showed generalized anxiety disorder, depressive symptoms, post-traumatic stress disorder (PTSD), sleep disturbances and they attributed their individual psychological distress to the COVID-19 pandemic. This patients received psychological/psychotherapeutic treatment due to the COVID-19 crisis, during hospitalization and after discharge.

**Conclusions:**

During the pandemic, patients with dermatological diseases needs not only medical support but also psychological and psychosocial support. This study results emphasize the importance of monitoring the psychological impact of the COVID-19 pandemic on patients with cutaneous manifestations.

**Disclosure of Interest:**

None Declared

